# Innovative Target Therapies Are Able to Block the Inflammation Associated with Dysfunction of the Cholesterol Biosynthesis Pathway

**DOI:** 10.3390/ijms17010047

**Published:** 2015-12-30

**Authors:** Annalisa Marcuzzi, Elisa Piscianz, Claudia Loganes, Liza Vecchi Brumatti, Alessandra Knowles, Sabrine Bilel, Alberto Tommasini, Roberta Bortul, Marina Zweyer

**Affiliations:** 1 Department of Medicine, Surgery and Health Sciences, University of Trieste, Piazzale Europa 1, Trieste 34128, Italy; claudia.loganes@gmail.com (C.L.); bortul@univ.trieste.it (R.B.); zweyer@units.it (M.Z.); 2 Institute for Maternal and Child Health—IRCCS “Burlo Garofolo”, via dell’Istria, 65/1, Trieste 34137, Italy; elisa.piscianz@gmail.com (E.P.); liza.vecchibrumatti@burlo.trieste.it (L.V.B.); alessandra.knowles@burlo.trieste.it (A.K.); alberto.tommasini@burlo.trieste.it (A.T.); 3 Cluster in Biomedicine (CBM scrl), Trieste 34128, Italy; sabrouna14@live.com

**Keywords:** cholesterol, mitochondria, apoptosis, autophagy, inflammasome

## Abstract

The cholesterol pathway is an essential biochemical process aimed at the synthesis of bioactive molecules involved in multiple crucial cellular functions. The end products of this pathway are sterols, such as cholesterol, which are essential components of cell membranes, precursors of steroid hormones, bile acids and other molecules such as ubiquinone. Several diseases are caused by defects in this metabolic pathway: the most severe forms of which cause neurological involvement (psychomotor retardation and cerebellar ataxia) as a result of a variety of cellular impairments, including mitochondrial dysfunction. These pathologies are induced by convergent mechanisms in which the mitochondrial unit plays a pivotal role contributing to defective apoptosis, autophagy and mitophagy processes. Unraveling these mechanisms would contribute to the development of effective drug treatments for these disorders. In addition, the development of biochemical models could have a substantial impact on the understanding of the mechanism of action of drugs that act on this pathway in multifactor disorders. In this review we will focus in particular on inhibitors of cholesterol synthesis, mitochondria-targeted drugs and inhibitors of the inflammasome.

## 1. The Cholesterol Pathway: A Pleiotropic Biochemical System

The cholesterol pathway (CP), also called the mevalonate pathway, is a crucial metabolic process that leads to the synthesis of cholesterol and other biomolecules such as steroidal hormones and isoprenoids. These essential bioactive molecules play an important role in multiple cellular processes, including intracellular signaling, gene expression, protein modification, cell growth/differentiation, cytoskeletal dynamics and stability, mitochondrial function and cell membrane structure [1,2].

The CP is promoted by a molecule of acetyl-CoA and its thiolase (Acetoacetyl-CoA), using the 3-hydroxy-3-methylglutaryl-Coenzyme A (HMG-CoA) synthase, to synthesize HMG-CoA. HMG-CoA reductase (HMGCR) then converts HMG-CoA to mevalonate (MVA), which is further metabolized to Isopentenyl-5-pyrophosphate (IPP) and its isomer Dimethyllallyl-pyrophosphate (DMAPP). At this point, Farnesyl pyrophosphate (FPP) synthase catalyzes a sequential reaction to generate mevalonate-derived isoprenoids, such as FPP and geranylgeranyl pyrophosphate (GGPP) [1,2,3].

For the synthesis of cholesterol, two molecules of FPP are converted by squalene synthase to the linear hydrocarbon molecule squalene, which is cyclized to the first sterol intermediate, lanosterol, and then converted, through a series of reactions, to cholesterol.

FPP is also one of the precursors of important metabolites such as dolichols, ubiquinones (Coenzyme Q), and carotenoids. These molecules are required for protein *N*-glycosylation (dolichols), mitochondrial electron transport chain function (ubiquinone), and free radical scavenging [1].

### HMG-CoA Reductase Controls the Cholesterol Pathway

The cholesterol pathway is essential for several cell functions. The regulation of this biochemical process has been intensely investigated and, in particular, the role of HMGCR which is the rate-controlling enzyme of cholesterol biosynthesis. This enzyme, ubiquitously expressed in all cells, is highly regulated and is controlled by a variety of mechanisms [4]. One of these is the negative feedback, a multivalent process by which cholesterol and isoprenoid products act as inhibitors of the reductase inducing its degradation from the membranes of the endoplasmic reticulum (ER) [5]. Furthermore, sterols and non-sterol metabolites control the transcription and the translation of HMGCR by reducing the amount of mRNA in response to increased levels of cholesterol [6].

In addition to the complex feedback mechanism, HMGR controls cholesterol levels through cross-regulation: this process is employed when the catalytic domain of HMGCR is inactivated through phosphorylation by an adenosine monophosphate-dependent kinase that alters the enzyme’s kinetic properties. It also occurs in response to invading pathogens or toxins that cause increases in HMGR mRNA levels and, thus, higher enzyme activity [7].

The regulation of HMGR is necessary for appropriate cholesterol synthesis. Defects in this enzyme lead to the development of inflammatory disorders [8] and diseases such as hypercholesterolemia [9]. Recent studies have proven that genetic errors can cause mutations in enzymes involved in the cholesterol cascade [10]; further investigations are necessary to determine the link between mutated enzymes and inflammatory phenotypes, in order to develop new therapies blocking the cholesterol damage in its early stages.

## 2. Diseases Linked to the Deregulation of the CP

Deregulation of the CP causes diseases that are severe and mostly monogenic. Among these, the mevalonate kinase deficiency (MKD) is a rare autosomal recessive disease caused by a blockade of the CP [11,12]. A defect in the pre-squalene activity of mevalonate kinase (MK, encoded by the mevalonate kinase gene, *MVK*) induces periodic fever syndromes, with different degrees of severity depending on the residual activity of mevalonate kinase: the autoinflammatory hyper immunoglobulinemia D (MIM 260920) is characterized by a 1%–8% residual MK activity, while in mevalonic aciduria (MIM 610377) MK level activity is undetectable [13,14].

There are several other disorders: Smith–Lemli–Opitz syndrome (MIM 270400), Conradi–Hünermann–Happle syndrome (MIM 302960) congenital hemidysplasia with ichthyosiform erythroderma and limb defects (CHILD, MIM 308050), and at least three extremely rare autosomal recessive disorders, Greenberg skeletal dysplasia (MIM 215140), lathosterolosis (MIM 607330) and desmosterolosis (MIM 602398). These syndromes show significant clinical overlap distinguished by physical and behavioral abnormalities, including nervous system dysfunctions with different degrees of severity. [10] (Table 1).

**Table 1:** Diseases involved in the deregulation of cholesterol pathway.

Disease/Syndrome	MIM	Genetics	Gene(s) Involved	Protein Involved	Molecular Features	Main Clinical Features
Mevalonate Kinase Deficiency (MKD) ^‡^ [15,16]	#260920 #610377	Autosomal recessive	*MVK*	Mevalonate kinase	Accumulation of mevalonic acid in urine and plasma	Elevated serum IgD/IgA, periodic fever, vomiting, diarrhea, psychomotor retardation, developmental delay, cerebellar and cerebral atrophy
Smith Lemli Opitz Syndrome (SLOS) [17,18,19,20]	#270400	Autosomal recessive	*DHCR7*	7-dehydrocholesterol reductase	Low cholesterol levels, accumulation of 7-DHC	Failure to thrive, microcephaly, micrognathia, ambiguous genitalia, limb shortening, polydactyly, mental retardation
Conradi-Hunermann-Happle [19,21]	#302960	X-linked dominant	*EBP*	Sterol-Δ8–Δ7-isomerase	Increased levels of 8-dehydrocholesterol and 8(9)-cholestenol	Growth deficiency, asymmetric limb shortening, mental retardation, ventriculomegaly
CHILD syndrome [19,22,23]	#308050	X-linked dominant	*NSDHL*	Part of the C-4 sterol demethylase protein complex	Increased levels of 8-dehydrocholesterol and 8(9)-cholestenol	Prenatal growth deficiency, hearing loss, unilateral distribution of abnormalities, skin lesions, erythema, severe skeletal abnormalities
Greenberg skeletal dysplasia [19,24]	#215140	Autosomal recessive	*LBR*	3β-hydroxysteroid-Δ14-reductase	Elevated cholesta-8,14-dien-3-β-ol in cultured fibroblasts and cholesta-8,14,24-trien-3β-ol in cartilage	Hydrops-ectopic calcification-moth-eaten (HEM) skeletal dysplasia, fetal death
Lathosterolosis [19,25]	#607330	Autosomal recessive	*SC5DL*	3β-hydroxysteroid-Δ5-desaturase	Increased levels of lathosterol in plasma and cultured fibroblast; absent 7-dehydrocholesterol, normal cholesterol	Microcephaly, polysyndactyly, colestatic liver disease, conductive deafness, severe psychomotor retardation
Desmosterolosis [19,26,27,28]	#602398	Autosomal recessive	*DHCR24*	3β-hydroxysterol-Δ24-reductase	Accumulations of desmosterol in plasma, kidney, liver, brain	Failure to thrive, microcephaly, anomalous pulmonary venous drainage, ambiguous genitalia, short limbs, generalized osteosclerosis, delayed psychomotor development, severe spasticity

^‡^ Table 1. List of disorders triggered by alterations on cholesterol pathway. Mutations in the *MVK* gene cause MKD that range from Hyper-IgD syndrome to Mevalonic aciduria, depending on the type and severity of the mutations.

## 3. Convergent Pathogenic Mechanisms on Deregulation of the Cholesterol Pathway

**Figure 1:**
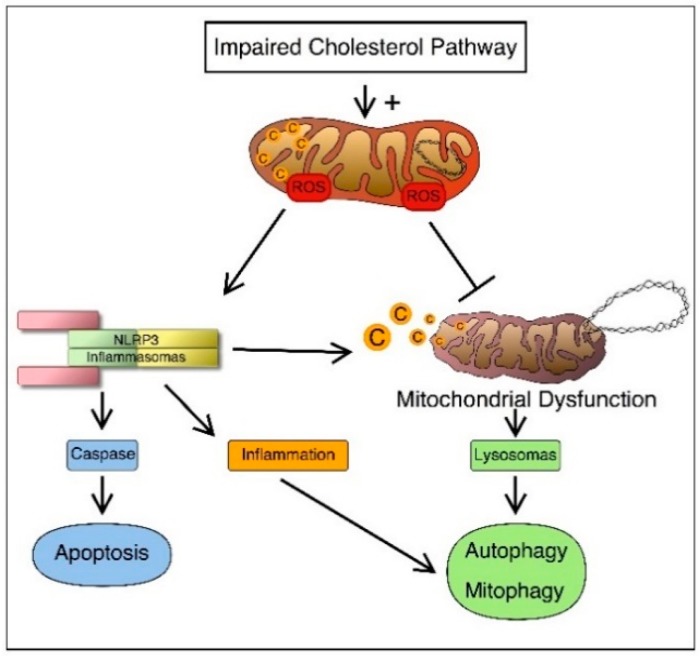
Connection between cholesterol disorders and inflammation. Once the cholesterol pathway is impaired, it can induce activation of the inflammosome and trigger cell apoptosis. On the other hand, production of (reactive oxygen species) ROS causes defective autophagy and/or mitophagy of damaged cells and organelles and this can further lead to NLRP3 (inflammosome) activation.

Despite progress in the understanding of the genetic causes that determine a number of pathologies associated with the deregulation of the CP, effective and definitive drug treatments are not always identifiable [20]. Defects in the mevalonate pathway lead to the activation of an inflammatory process and of cellular mechanisms such as programmed cell death linked to mitochondrial damage, autophagy and mitophagy (Figure 1).

### 3.1. Inflammatory Mechanisms

The deregulation of the CP implies the activation of an inflammatory process by specific multiproteins, called inflammasomes [29]. Inflammasomes, described by Martinon and colleagues [30], are large molecular platforms, specialized in recognizing danger signals and in instructing the general defense mechanisms of the innate immune system [30,31,32]. To date, a number of inflammasomes have been clearly identified [33]: Nucleotide-binding oligomerization domain, Leucine rich Repeat and Pyrin domain containing (NLRP)1 [34], NLRP2 [35], NLRP3 [36], NLRP6 [37], NLRP7 [38], NLRP12 [39], NLR apoptosis inhibitory protein(NAIP)/NLR family, CARD-containing 4 (NLRC4) NAIP/NLRC4 [40]. The NLRP3 protein, in particular, is the best characterized component in the inflammasome platform and has been shown to be implicated in the development of chronic diseases. NLRP3, which is a crucial interface between metabolism and inflammation [41], is induced by a wide spectrum of molecules through mechanisms that have not, as yet, been fully understood.

A variety of models have been proposed to explain the main pathway of activation of NLRP3: there are interesting data showing a crucial involvement of reactive oxygen species (ROS), produced by damaged mitochondria [42]. However, the mechanism underlying the role of ROS in priming NLRP3 remains unclear [43]. Another model, designed by Hornung and colleagues, suggests that the NLRP3 inflammasome could be triggered by phagolysosomal destabilization and lysosomal damage: this “endogenous danger signal” represents for the immune system the cause activing the inflammasome [44].

In the innate immune response, an abnormal synthesis of ROS could be associated with decreased bioavailability of nitric oxide (NO), which is a major indicator of NLRP3 activation. Indeed, the inhibition of NLRP3 activation by NO is known to be one of the mechanisms of tissue protection by ischemic preconditioning [45].

ROS and NO have been proposed as triggers of mitochondrial dysfunction [46,47,48], and data in the literature have shown that cardiovascular disorders [49] and metabolic syndromes that are related to cholesterol deregulation are associated with mitochondrial damage [50,51,52,53,54,55].

**Figure 2:**
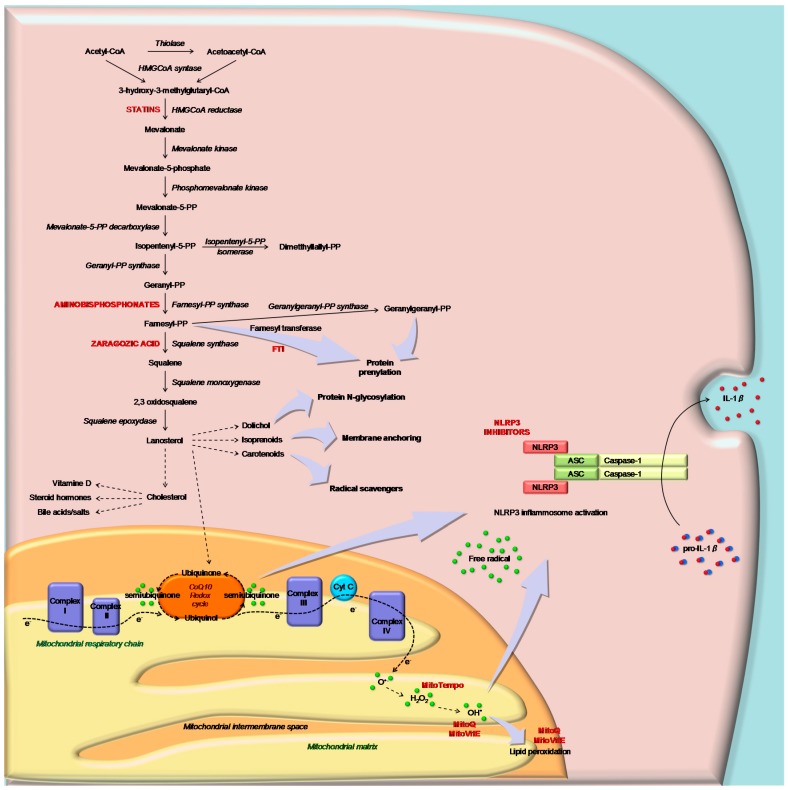
Schematic representation of the cholesterol pathway and of the mediation of sterol metabolites (ubiquinone) in mitochondrial respiratory function (Complex I–IV) in response to the inflammation signal. Inhibitors of CP and MTAse (as MitoQ) are indicated in capital and red characters: statins, aminobisphosphonate, squalene synthase and farnesyltransferase inhibitors. The block of CP leads to the activation of the NLRP3 inflammasome and consequently IL-1β through mitochondrial signals.

Several recent data have shown that autophagy [56], and in particular mitophagy, are key links between inflammasome ROS and mitochondrial dysfunction [57,58] (Figure 2).

### 3.2. Programmed Cell Death

Programmed cell death or apoptosis is a homeostatic process that maintains cell populations in tissues and acts as a defense mechanism against intracellular damage caused by infection or oxidative stress. This mechanism is essential for optimal cell function and for the development of immune and hormonal systems [59]. Defects of apoptosis cause severe diseases such as cancer, Alzheimer’s, Parkinson’s and autoimmune conditions [59]. Programmed cell death, linked to an inflammatory response by the NLRP3 inflammasome, is triggered by the mitochondrial oxidative stress induced by an increase of ROS, and is followed by the apoptosis mechanism initiated by caspases [60,61]. The mitochondria play a central role in activating and controlling cell death mechanisms through caspase activity [60]. Our studies on MKD pathogenesis confirm that CP deregulation induces programmed cell death via mitochondrial signals, especially in neurological areas [62,63].

### 3.3. Autophagy and Mitophagy

Autophagy is a catabolic process with multiple physiological functions, which acts primarily as a protective mechanism to prevent cell death. A specific type of autophagy, called mitophagy, is designed to ensure the integrity of healthy mitochondria and the removal of damaged intracellular structures [64,65], preventing the accumulation of toxic mitochondrial products. The impairment of this mechanism contributes to the accumulation of ROS and to the synthesis of pro-inflammatory molecules that trigger programmed cell death. In addition to this, the abnormal biosynthesis of ROS supports a hyper-activation of the inflammatory pathways, e.g., though the NLRP3 inflammasome [66].

Low levels of activation of mitophagy may be involved in the pathogenesis of common human disorders such as Parkinson disease, diabetes, Alzheimer’s disease and some forms of cancer [67,68].

Understanding the molecular mechanism of mitophagy is, therefore, of crucial importance to explain its pivotal role in cellular homeostasis and to comprehend how altered ”mitodynamics”, (e.g., aberrant mitochondrial trafficking, modified interorganellar communication, and impaired mitochondrial quality control) may contribute to the onset of numerous diseases, from inborn errors of cholesterol biosynthesis to adult-onset neurodegenerative diseases, cancer, cardiovascular disorders, and infectious/inflammatory conditions, as well as metabolic derangements [69].

Furthermore, impaired autophagy, controlled by multiple pathways including protein isoprenylation, has been identified as one of the causes of the pathogenesis of SLOS [70], due to inefficient clearance of defective mitochondria. Similarly, in the pathogenesis of MKD, the cumulative effect of mitochondrial deregulation triggers the activation of NLRP3 and, consequently, the biosynthesis of pro-inflammatory cytokines that amplify the inflammation system [71,72,73].

## 4. Innovative Target Therapies Counteracting Inflammation Caused by Cholesterol Dysfunctions

In the light of the pathogenic mechanisms of CP deregulation, the potential therapeutic targets are both at pathway level (activation of NLRP3) and at molecular level (mitochondria and inflammasome) (Figure 2).

### 4.1. Inhibitors of Cholesterol Synthesis

The cholesterol pathway cascade is regulated by, among others, drugs such as statins (mycotoxins used to lower cholesterol levels), and bisphosphonates, used to treat various bone-degenerative diseases, including osteoporosis. These two agents, which are widely used in clinical practice for their structural resemblance to substrates within the pathway, can inhibit mevalonate synthesis (statins) or mevalonate downstream metabolism (bisphosphonates), leading to reduced formation of the isoprenoids FPP and GGPP [73,74].

The statins are a class of drugs similar to HMG-CoA in chemical structure that inhibits HMGCR, binding competitively to the active site of the enzyme. This competition reduces mevalonate biosynthesis and slows down the subsequent serial steps to produce cholesterol. According to Williams (2002), statins have greater affinity for HMGCR than HMG-CoA, therefore they are particularly efficient in controlling the production of excessive levels of cellular cholesterol [75]. Depending on dosage and type, statins display differential capacity to inhibit the mevalonate pathway and to prevent coronary and atherosclerosis complications [76]. For all these reasons, they are considered the “gold standard” for metabolic prevention of coronary heart diseases [77].

Statins are divided into two categories based on their molecular structure and chemistry: natural statins, such as lovastatin, simvastatin, and pravastatin, and synthetic statins, such as fluvastatin, cerivastatin, atorvastatin, and rosuvastatin [78].

The chemical composition influences the pharmacological properties of these drugs, affecting, for example, their affinity for the enzyme HMGCR, their systemic availability and their metabolism/excretion pathways [79].

A variety of combinations of statins with other agents, such as ezetimibe/simvastatin, are available on the pharmaceutical market [80].

Despite the widespread use and high safety profile of statins, recent studies have produced controversial data regarding the side effects of these drugs in particular myopathy and rhabdomyolysis [81,82]. Indeed, a recent observation has suggested that these adverse clinical effects may be caused by the mitochondrial dysfunction caused by statins [82]. According to this study, these drugs are able to promote mitochondrial permeability transient pore opening and generate apoptotic proteins, but no changes in HMGCR activities have been noted. However, because of the contradictory results and interpretations in literature, further studies are necessary to verify this hypothesis.

Finally, Izadpanah proposed a very fitting theory on these adverse clinical effects, suggesting that statins play a role in inflammation pleiotropy through stem cells: the positive effect is supported by the capacity of mesenchymal stem cells to differentiate into macrophages capable of reducing inflammation, while the negative effect could be determined by an increase in cellular senescence and apoptosis markers [83].

Bisphosphonates (BPs) are another family of drugs that act on the CP: they are currently employed to treat pathological conditions characterized by osteoclast-mediated bone loss, due to osteoporosis and other metabolic bone diseases (osteolytic bone metastasis, hypercalcemia and Paget’s disease). BPs have the ability to inhibit bone digestion and resorption by concentrating bone phosphates and inhibiting osteoclast function and viability, thereby slowing down bone loss [84]. They are chemically stable analogs of pyrophosphates and compete with the analogue portion of the isoprenoid diphosphate intermediates of the mevalonate pathway. The impact of BPs on the CP is mainly due to their pyrophosphate-like structure, which enables them to bind to, and inhibit, key isoprenoid biosynthetic enzymes, such as farnesylpyrophosphate synthase (FPPS). The inhibition caused by the structure of BPs prevents the post-translational prenylation of various Guanosine-5′-Triphosphate (GTP)-binding proteins, not only in osteoclast, but also in blood cells, leading to an impairment of the various cellular functions these proteins modulate [85,86].

BPs can be divided into two groups, nitrogen-containing (*N*-containing) and non-*N*-containing BPs (or pyrophosphate-resembling BPs), based on their different mechanism of action and cell effects [73,74].

The former class has become available more recently and is more powerful. It includes alendronate, pamidronate, risedronate, ibandronate and zoledronate and interferes with the mevalonate pathway by blocking FPPS. The second group, which includes etidronate and clodronate, interferes with adenosine triphosphate (ATP)-dependent intracellular pathways: the non-*N*-containing BPs are metabolically incorporated into nonhydrolyzable ATP analogues that disturb mitochondrial activity and inhibit ATP-dependent enzymes [87,88,89].

Also in the case of BPs there are data in the literature describing effects that are exactly opposite to those illustrated above [90]. The main side effects related to the use of these drugs are the following: renal toxicity [91,92] and acute-phase reactions (related specially to zoledronic acid) [93,94,95,96,97], gastrointestinal toxicity (associated to oral agents as clodronate and ibandronate) [98], and osteonecrosis of the jaw as an adverse effect of the aminobisphophonate therapy in patients whose immune system is already compromised [99].

In addition, a number of plant and fungal derived isoprenoids and inhibitors, introduced in various amounts through the diet, are able to modulate the CP. For example, natural isoprenoid compounds, such as geraniol, menthol, farnesol, profoundly affect MP regulation, as also do squalene synthase inhibitors (zaragozic acid, ZAA) [100,101] and inhibitors of farnesyltransferase (manumycin A, Tipifarnib) [102,103].

### 4.2. Mitochondrial-Target Anti-Oxidants

The drugs that belong to the Mitochondrial-Target Anti-oxidants (MTAs) family are studied for their anti-oxidant activity and mitochondrial protection properties, and are therefore good candidate molecules to contrast oxidative stress caused by cholesterol deregulation [104]. MTAs are mitochondria-targeted drugs several hundred-fold more potent at preventing mitochondrial oxidative damage than untargeted antioxidants [90]. Once accumulated in the mitochondria, MTAs are able to inhibit selected steps along the pathway of activation of programmed cell death [105,106]. The compounds that belong to the MTAs family are MitoQ, Mitotempo and MitoVitE. MitoQ, in particular, is a promising antioxidant biomolecule that has been shown to protect mitochondria from various types of oxidative damage by decreasing ROS production [106,107,108,109,110,111,112,113,114,115,116,117,118,119].

The antioxidant component of MitoQ is ubiquinone, the same bioactive molecule found in Coenzyme Q10 [108].

MitoQ, positively charged with a lipophilic cation, is able to penetrate the biological membranes and accumulate selectively in the mitochondria, within the negative mitochondrial matrix [119]. The mitochondrial respiratory chain rapidly reduces MitoQ ubiquinone to its active ubiquinol form, MitoQuinol, which acts as antioxidant molecule and mobile electron donor [107,119]. In particular, ubiquinol decreases local oxidative damage, by donating a hydrogen atom to radical species formed during lipid peroxidation [120,121,122].

After neutralizing a free radical or ROS, the ubiquinol form is converted to ubiquinone, which is subsequently converted back to the active ubiquinol form [108,123], with restored antioxidant function.

Recently, the mitochondrial effects of MitoQ have been tested in several *in vitro* models demonstrating that this Mitochondrial-Target drug can reduce free radicals and oxidative damage, maintaining mitochondrial function and preventing cell death caused by endogenous oxidative stress [106,111,124,125].

The MitoQ antioxidant mechanism may be due to the inhibition of lipid peroxidation in the mitochondrial inner membrane, but further studies are necessary to define the specific mechanism of action of this drug in preventing mitochondrial damage [126].

Mito Q is already being studied as potential treatment for neurodegenerative diseases such as Alzheimer’s disease and ischaemia-reperfusion injury, as well as other ageing-related dysfunctions [107].

Potentially therapeutic concentration of MitoQ could be delivered to mitochondria *in vivo* through the oral administration of well-tolerated doses of the drug [127].

MitoQ has been shown to decrease mitochondrial damage even at high doses in rats, without side effects [128]. Data in the literature have provided robust evidence to support the testing of MTAs in preclinical trials using neurodegenerative disease cell and mouse models, as well as on Alzheimer’s patients.

The use of mitochondria-targeted peptides has also been shown to be effective in treating/reducing hypercholesterolemia in an animal model of acute kidney injury, in which renal injuries appeared to be alleviated after peptide treatment [129]. Data in literature confirm that several diseases, including cardiovascular and neurodegenerative diseases, insulin resistance and diabetes as well as age-related cancers, bear a common relationship to mitochondrial damage; this would support the hypothesis of a pivotal role for MTAs [130].

### 4.3. NLRP3 Inhibitors

Although this issue is still controversial, some data link the CP to the NLRP3 inflammasome. MKD, for example, is associated with an autoinflammatory phenotype which is probably linked to reduced protein prenylation and/or increased mitochondrial damage. Whatever the mechanism involved, autoinflammation is associated with increased activation of NLRP3 and Caspase-1. Thus, inhibition of NLRP3 may deserve to be further investigated for its potential to deal with inflammatory phenomena related to CP dysfunction. Over the past 10 years, a growing body of data has been collected regarding the function of NLRs, which is crucial for the identification of new therapeutic opportunities using small molecule inhibitors [130,131].

A new class of small molecule inhibitors of NLRP3 has recently been discovered. Coll and colleagues have described an innovative compound, called MCC950, which effectively reduces the synthesis of IL-1β in a disease model of multiple sclerosis [132], and could represent an effective tool to treat NLRP3-associated syndromes, such as autoinflammatory and autoimmune diseases [133]. Another compound, known as Andro, was tested in an experimental design *in vivo* and in an *in vitro* model of colon carcinogenesis, with the aim of establishing the effect of the NLRP3 inflammasome. It is worth noting that Andro acts as a trigger, promoting mitophagy in macrophages by inducing a decrease in membrane potential through the inactivation of the NLRP3 inflammasome [134].

The interest elicited by the effectiveness of this new class of drugs is supported by a growing body of data. Following the synthesis of compounds such as glyburide [135], which was the first molecule to be studied for its ability to prevent NLRP3 activation, albeit only at high doses *in vivo*, a number of similar molecules has been synthesized. Among these, 16673-34-0 (5-chloro-2-methoxy-*N*-[2-(4-sulfamoylphenyl)ethyl] benzamide), which was developed to limit the infarct size following myocardial ischemia–reperfusion in a mouse model, proved to be highly effective [136].

## 5. Outstanding Questions

Several studies have described how the oxidative damage of mitochondria and the activation of inflammasomes can interact to determine defects of the CP. Targeting these two mechanisms would be relevant both to curing rare disorders of cholesterol biosynthesis and to the treatment of metabolic and degenerative disorders. Although considerable evidence, deriving from cellular and animal models, is available, much remains to be understood about the real potential of inflammasome inhibitors in the clinical setting. The crucial fact remains that cholesterol is tightly regulated by positive and negative feedback mechanisms that may affect different cell types and functions in different ways.

For example, consistent data show that shortage of geranygeranyiol can lead to inflammation by reducing prenylation of membrane bound GTP-binding proteins of the Rab and Rac family, with consequent activation of the inflammasome [137,138,139,140]. However, recent data support the idea that also the shortage of cholesterol may exert an anti-inflammatory effect by reducing the substrate available to synthesize 25-hydroxycholesterol [141]. The matter becomes even more complicated if we consider that 25-hydroxycholesterol can regulate the enzymes of the pathway through modulation of transcription factors, the so-called sterol regulatory element-binding proteins (SREBPs), and thus affect the concentration of mevalonate-derived compounds.

Furthermore, the regulation of the CP may vary greatly depending on the cell type. CP plays an essential role in liver metabolism and in the production of biliary acids. In monocytes, the main function of the CP is probably to modulate the activity of membrane bound proteins or regulate cell membrane composition. Therefore, it is difficult to predict how pharmaceutical interventions on this pathway may influence the various functions of the different cells types and organs.

For the same reasons, it is hard to predict what the long term consequences of treatments that reduce the inflammatory response and hyper-protect mitochondria could be. Further studies addressing these questions in preclinical settings are needed. In the meantime, randomized controlled trials will definitely clarify the preventive and curative potential of these novel therapeutic strategies on neurodegenerative and cardiovascular inflammatory disorders.
